# Genome-wide haplotype association study identify the FGFR2 gene as a risk gene for Acute Myeloid Leukemia

**DOI:** 10.18632/oncotarget.13631

**Published:** 2016-11-26

**Authors:** Hongchao Lv, Mingming Zhang, Zhenwei Shang, Jin Li, Shanshan Zhang, Duan Lian, Ruijie Zhang

**Affiliations:** ^1^ College of Bioinformatics Science and Technology, Harbin Medical University, Harbin, China; ^2^ Hospital of Harbin Turbine Company Limited, Harbin Electric Corporation, Harbin, China

**Keywords:** acute myeloid leukemia, haplotype, gene prioritization

## Abstract

Acute myeloid leukemia (AML) is a cancer of the myeloid line of blood cells, and generally considered to be caused by environment and genetic factors. In this study, we combined a genome-wide haplotype association study (GWHAS) and gene prioritization strategy to mine AML-related genetic affect factors and understand its pathogenesis. A total of 175 AML patients were downloaded from the public GEO database (GSE32462) and 218 matched Caucasian controls were from the HapMap Project. We first identified the linkage disequilibrium (LD) blocks and performed a GWHAS to scan AML-related haplotypes. Then we mapped these haplotypes to the corresponding genes as candidate. And finally, we prioritized all the AML candidate genes based on the similarity with 38 known AML susceptibility genes. The results showed that 1754 haplotypes were significant associated with AML (P<1E-5) and mapped to 591 candidate genes. After prioritizing all 591 AML candidate genes, we obtained four genes ranking at the front as AML risk genes: RUNX1, JAK1, PDGFRA, and FGFR2. Among them, RUNX1, JAK1 and PDGFRA had been confirmed as AML risk genes. In particular, we found that the gene FGFR2 was a novel AML susceptibility gene with a haplotype TT (rs7090018 and rs2912759) showed significant association with AML (P-value = 7.07E-06). In a word, our findings might provide a new perspective to understand the pathogenesis of AML.

## INTRODUCTION

Acute myeloid leukemia (AML) is one of the cancer in myeloid line of blood cells, characterized by clonal expansion of myeloid progenitors in the bone marrow and peripheral blood [1]. AML was the most common acute leukemia, and increased with the population ages [2]. In the USA alone, its incidence was 3.5 cases per 100,000 people, and approximately 20,000 patients were diagnosed with AML and 10,000 AML-related deaths occurred in 2015 [3]. AML with t(8;21)(q22;q22) [RUNX1-RUNX1T1] and inv(16)(p13.1q22) or t(16;16)(p13.1;q22) [CBFB-MYH11], which the aberrant fusion genes leaded to impaired differentiation of hematopoietic progenitors, commonly referred to as core-binding factor (CBF)–CAML. It accounted for approximately 15% of AML patients, and was considered to have relatively good prognosis compared to other leukemia subtypes [4]. In recent years, many studies had revealed novel mutations, epigenetic changes, and/or aberrant expression levels of protein-coding and noncoding genes involved in leukemogenesis. These results could help us to understand the genetic basis of the disease and refine the risk assessment in CBF-AML. Furthermore, they would serve as targets for novel therapeutic approaches [4, 5]. With the development of high-resolution genome-wide scanning technologies, we could detect more AML-related SNPs, copy number alterations (CNAs) and other regions in AML and other myeloid neoplasms.

Kühn MW, et al shared a genome-wide CBF-AML SNP-array data set with pediatric and adult CBF-AML patients (GSE32462). Based on the mutation analyses and CNAs analyses, they identified multiple activating mutations in KIT, FLT3, JAK2, NRAS, and KRAS, and revealed that in some cases, more than one gene within the RAS/Kinase signaling pathway were activated [4]. As we know, a sequence of linkage disequilibrium (LD) alleles on a particular chromosome region might make up of a haplotype block, which would contain genetic information of several SNPs with marginal effects. Comparing the results about single-marker analysis, several previous studies confirmed that haplotype analysis results could increase power for detecting associations with disease and were robustness for mapping disease genes [6, 7]. Thus we performed a genome-wide haplotype association analysis study (GWHAS) to identify potentially novel AML risk genes. The International HapMap Project is an important resource in the human genome research and provides a variety of adjacent populations as a reference. In view of there were not any normal individuals in GSE32462, we constructed a control samples set from the HapMap Project to effectively use the precious genome-wide SNPs data set. Furthermore, according to the related genes in a disease or biological process generally shared similar characteristics in multiple omics data resources [8, 9], a gene prioritization strategy was adopted to assess the similarity between AML candidate genes and known AML susceptibility genes.

## RESULTS

### The results of genome-wide haplotype association study

In this study, a total of 739,981 autosomal SNPs were included, which shared in both GPL6801 platform and the HapMap database. After the quality control (detail see methods), our study obtained a total of 734,624 eligible autosomal SNPs. Then a genome-wide haplotype association study was performed, which contained 393 samples (175 AML patients and 218 matched healthy individuals). We identified 118,057 haplotype blocks, which contained total 519,865 haplotypes. For each haplotype, we carried out a chi-square test to assess the relationship with AML risk. In the end, 1,754 haplotypes located on 1,089 block regions showed significant correlation with AML (P< 1E-5) (Supplementary Table 2).

### Mapping and prioritizing the AML candidate genes

According to the chromosome location information, 1,754 significant haplotypes were mapped to 591 genes, and regarded them as AML-related candidate genes. Meanwhile, we retrieved 38 known AML susceptibility genes from OMIM and GAD database, which had been confirmed to associate with AML risk. After that, we analyzed the similarity on the structure and function between two gene sets, and total 42 features were included, such as Ensembl Est, Gene Ontology, KEGG, Blast, HPRD, and so on (See methods for more detailed). We performed all above analyses by the Endeavour software. At last, four genes, RUNX1, JAK1, PDGFRA, and FGFR2, had lower P-value (P<0.05), and regarded as high-risk AML genes (Table 1). All detailed results for candidate genes prioritization could be seen in Supplementary Table 3.

**Table 1:** The AML related genes for prioritization according to reliability verification

Ensembl gene ID	Gene symbol	Gene description	location	P-value
ENSG00000159216	RUNX1	runt related transcription factor 1	21q22.3	0.0016
ENSG00000162434	JAK1	janus kinase 1	1p32.3-p31.3	0.0318
ENSG00000134853	PDGFRA	platelet derived growth factor receptor alpha	4q12	0.043
ENSG00000066468	FGFR2	fibroblast growth factor receptor 2	10q26	0.0494

Compared with the known AML genes, the candidate genes were ranked based on P-values of global prioritization. We listed the top four candidate genes with P-values lower than 0.05 in Table 1.

### The analysis of RUNX1 gene

**Figure 1:**
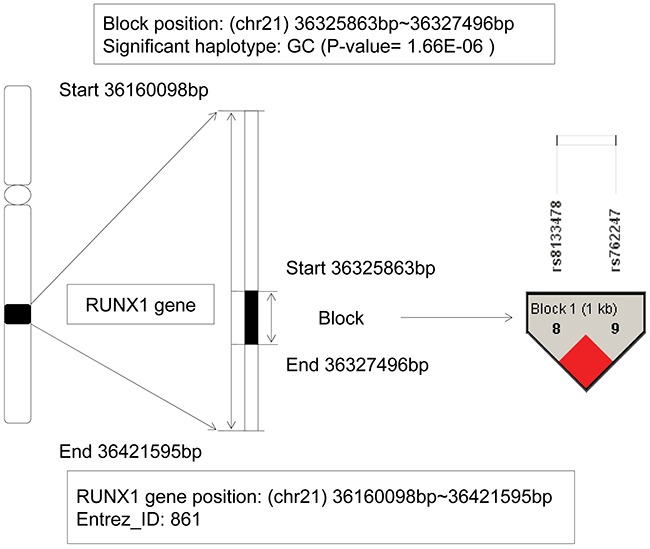
The haplotype analysis result of RUNX1 gene

The runt related transcription factor 1 (RUNX1) gene, also named as AML1, PEBP2, located on chromosome 21q22.3, and the physical location was: 36,160,098bp (start) – 36,421,595bp (end). The RUNX1 gene ranked the first, and also was the only gene existed in both training and candidate gene sets. From the detail information on Figure 1, we could see a LD block located on the gene with 2 SNPs: rs8133478 and rs762247, and a haplotype GC showed significant association with AML (P-value = 1.66E-6). Based on the EM algorithm to estimate the frequency of haplotype, it in patients was 5.95E-2, whereas in control individuals was 2.57E-3. Obviously, the haplotype GC could be inferred as a risk factor for AML with an OR=23.14. Generally, as a member of the RUNX gene family, it appeared frequently in the development of all hematopoietic cell types. The RUNX1 gene was involved in the t(8;21) translocation in acute and chronic myeloid leukemia, and could produce oncogenic transformation to AML [10]. It was also demonstrated associated with other types of leukemia [11, 12], such as frequently mutated in pediatric B progenitor acute lymphoblastic leukemia (ALL) in Pui (1995)'s research [13].

### The analysis of JAK1 gene

**Figure 2:**
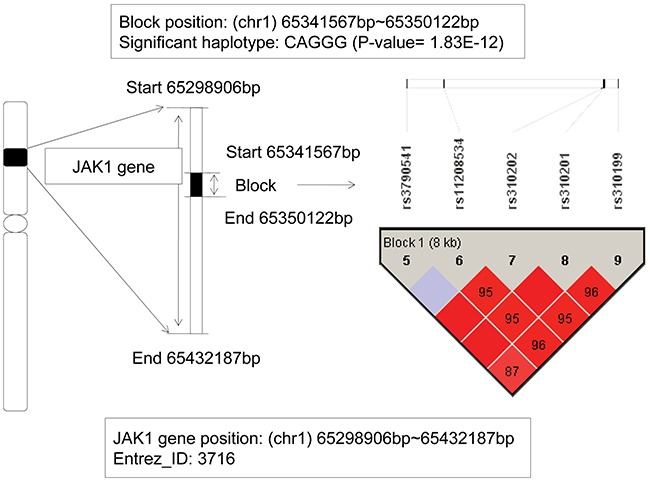
The haplotype analysis result of JAK1 gene

The janus kinase 1 (JAK1) gene located from 65,298,906bp (start) to 65,432,187bp (end) on chromosome 1p32.3-p31.3. The detailed information could be seen on the Figure 2. In this gene, we found a haplotype CAGGG showed significant association with AML (chi-square test p value is 1.83E-12), which located on a block that consisted of 5 SNPs: rs3790541, rs11208534, rs310202, rs310201, and rs310199. As the frequency of the haplotype CAGGG in patients was 0.118, and in control individuals was 2.57E-3, and the odds ratio was 45.8, we could infer that the haplotype CAGGG was an AML risk factor, and the individuals carried it might increase the genetic risk of AML. The oncogenic JAK1 gene encoded a cytoplasmic tyrosine kinase and involved in lymphoid cell precursor proliferation, survival, and differentiation [14]. Dysregulation of JAK-STAT pathway had been found as key events in a variety of hematological malignancies [15]. Additionally, it has been reported that somatic mutations in JAK1 occurred in individuals with acute lymphoblastic leukemia (ALL) and the dysregulated JAK1 function effected on ALL, particularly of T cell origin [14, 16]. Furthermore, Xiang Z–s report firstly demonstrated somatic JAK1 mutations in AML and suggested that JAK1 mutations might have function as disease-modifying mutations in AML pathogenesis [17]. According to the association analysis about the regions of strong LD, our study further revealed that the JAK1 gene might be an AML-related risk gene.

### The analysis of PDGFRA gene

**Figure 3:**
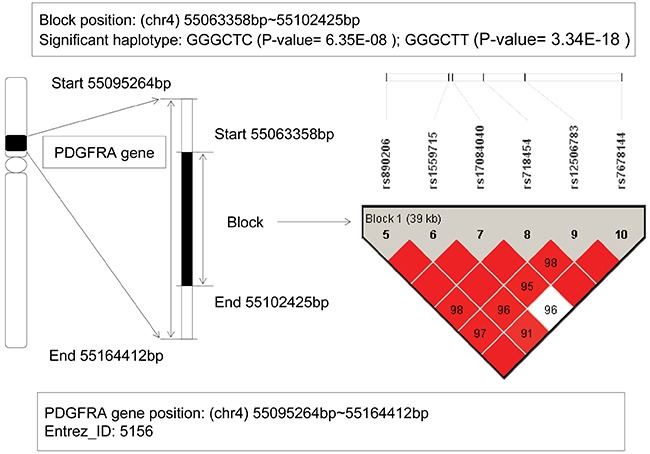
The haplotype analysis result of PDGFRA gene

From our GWHAS, a LD block was found on the platelet derived growth factor receptor alpha (PDGFRA) gene with a physical location from 55,063,358bp to 55,102,425bp. Among six haplotypes, two haplotypes GGGCTC and GGGCTT showed significant association with AML (P-value = 6.35E-08, and 3.34E-18) (see Figure 3). For the haplotype GGGCTC, it can be inferred as a protective factor with an OR=0.284, and however, the haplotype GGGCTT showed a risk effect with OR=841.67. Studies revealed that this gene involved in organ development, wound healing, and tumor progression. Mutations in the PDGFRA gene had been implicated in idiopathic hypereosinophilic syndrome [18], somatic and familial gastrointestinal stromal tumors [19], brain tumor [20] and a variety of other cancers. Hiwatari M's study revealed [21] novel missense mutations in the tyrosine kinase domain of the PDGFRA gene in childhood acute myeloid leukemia with t(8;21)(q22;q22) or inv(16)(p13q22). The results suggested that PDGFRA mutations might be implicated in oncogenic mechanisms in AML. Moreover, recent studies showed if the eosinophilia-associated AML patients presented the FIP1L1-PDGFRA fusion gene, they should be as excellent candidates for treatment with tyrosine kinase inhibitors [22]. In our study, the relationship between PDGFRA gene and AML risk was detected again.

### The analysis of FGFR2 gene

**Figure 4:**
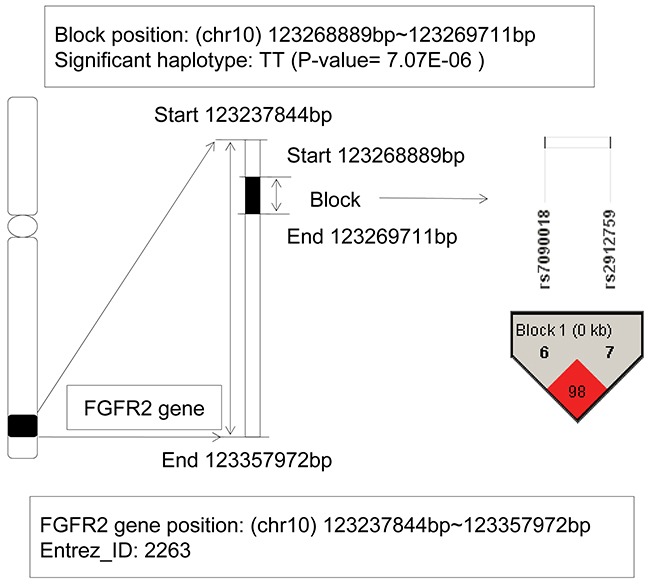
The haplotype analysis result of FGFR2 gene

The fibroblast growth factor receptor 2 (FGFR2) gene located on chromosome 10q26 with the physical location: 123,237,844bp (start) – 123,357,972bp (end). From Figure 4, a LD block contained 2 SNPs: rs7090018 and rs2912759, located on this gene, and a haplotype TT showed significant association with AML (P-value = 7.07E-06). When we implemented the haplotype frequency estimation via the EM algorithm, the frequencies of the haplotype TT were 0.0463 in patients and only zero in control individuals, respectively. Although the allele frequency in patients showed relatively low, the haplotype TT still revealed a risk effect compared to the lower control group. In recent years, the association between mutations in FGFR2 gene and various cancers had been found, such as breast cancer [23], endometrial cancer [24], oral squamous cell carcinoma [25], gastric cancer [26], and so on. Further, fibroblast growth factor receptors (FGFRs) genes had been shown to be translocated in multiple myeloma (MM) and myeloproliferative disorder (MPD) [27, 28]. Jang JH, et al's study also found a splice variant of FGFR2 gene in human leukemia HL-60 cells, and revealed that it was associated with AKT and MAPK pathway activation [29]. All of these indicated its oncogenic potential. In additional, Tanizaki J, et al's study identified a novel FGFR2 extracellular domain insertion mutation and demonstrated that they were both oncogenic and sensitive to inhibition by FGFR kinase inhibitors [30]. From the perspective of linkage disequilibrium, our study for the first time revealed the relationship between the FGFR2 gene and AML risk.

## DISCUSSION

Acute myeloid leukemia was recognized as a complex disease of hematopoietic stem cell disorders. It was classified into several categories based on underlying genetic alterations to facilitate diagnosis and prognosis by the World Health Organization (WHO). Among them, CBF-AML was characterized by the presence of distinct cytogenetic abnormalities [31]. The genetic alterations discovered in CBF-AML would help us to understand the process of leukemogenesis and also serve as targets for novel therapeutic approaches [32]. Although its pathogenesis mechanisms remained unclear, new molecular technologies had allowed for in-depth molecular analyses of AML patients and revealed some molecular aberrations such as novel mutations, epigenetic changes, and so on [5]. Moreover, the LD region on a gene could be regarded as a sensitive indicator of the population genetic forces [33]. Based on the genotyping arrays dataset GSE32462, we firstly performed a GWHAS to identify the AML related haplotypes. It covered the marginal effects of SNPs in a haplotype block and might have higher efficiency to mined AML susceptibility genes [6]. Here, a total of 1,089 block regions were caught, and mapped to 591 AML candidate genes. Then based on multi-omics data resources, we prioritized and identified 4 AML risk genes: RUNX1, JAK1, PDGFRA, and FGFR2. Among of them, RUNX1 ranked the first and was an oncogene for AML; JAK1 and PDGFRA gene also were confirmed association with AML. Through retrieving the published literature, our study revealed that FGFR2 gene, which had been involved in various cancers, was association with AML risk for the first time.

Recent years, the development of high-resolution genome-wide scanning technologies had given us abundant genetic variation resources for mining disease pathogenesis. Although still a lot of work need to be done, we believe that our work is effective and feasible. The study about conserved linkage disequilibrium chromosome regions can provide a new perspective for subsequent understanding of the pathogenesis about AML.

## MATERIALS AND METHODS

### Datasets and Quality control

In this study, we downloaded all the original genotypes data of Caucasian CBF-AML patients from GEO database (accession GSE32462) under the chip platform GPL6801 (Affymetrix SNP 6.0 array), which contained 930,662 SNP markers. The purpose of our study was to identify the genetic risk factors of acute myeloid leukemia and avoid the effect of acquired somatic mutation. Therefore we selected the germline DNA of 175 pediatric and adult CBF-AML patients obtained from bone marrow or blood at remission. Considering the International HapMap Project could provide a variety of geographically neighboring reference populations, we compared the similarity of allele frequencies between HapMap Caucasian populations and GSE32462, and selected the highest correlation the HapMap CEU and TSI populations as a reference. Finally, 218 unrelated Caucasian control individuals were downloaded from phase II+III of the International HapMap Project (CEU, 116; TSI, 102,ftp://ftp.ncbi.nih.gov/hapmap) [34, 35]. We took them as normal control samples. There were 739,981 autosomal SNPs shared in both GPL6801 platform and the HapMap database. Then we performed a quality control (QC) to select data according to the following criteria: Hardy–Weinberg equilibrium (HWE) P > 0.001, minor allele frequency (MAF) > 0.001, and percentage of successfully genotyping for the marker >75%. Finally, a total of 734,624 autosomal SNPs passed the QC and were used in the subsequent analyses.

### Genome-wide haplotype association analysis

On the basis of the autosomal SNPs data of 393 case-control samples, we performed a GWHAS to identify AML-related haplotypes. First, we used the Four Gamete Tests (FGT) method to assess the linkage disequilibrium (LD) blocks [36]. Then the haplotype phasing was estimated using the Maximum Likelihood Estimation (MLE) and the frequencies of the haplotypes were estimated using the Expectation Maximization (EM) algorithm. Finally, a chi-square test was performed, and if the P-value less than 1E-5, the haplotype was captured and considered as an AML-related candidate. All above analyses were done using the HAPLOVIEW software [37].

### Mapping AML candidate genes

We mapped all the AML-related candidate haplotypes to genes based on the chromosome location information. All the genes location information was queried from the NCBI gene database. If a gene shared the same chromosome fragment with at least one AML candidate haplotype, it would be regarded as an AML candidate gene.

### Prioritizing candidate genes to find AML risk genes

In some ways, the genes involved in the defined biological processes or diseases often shared some related characteristics, such as the similarity for sequence, functional annotation, expression and regulation information [9, 38]. Therefore, it could be considered that the AML potential risk genes should share some genetic features with the known AML genes either directly or indirectly. In order to identify the AML risk genes, we adopted a gene prioritization strategy proposed by Aerts S, et al [9]. We performed it in three simple steps. First of all, we used the known disease genes as a training set, and established the training models based on the biological process of interest. Each sub-model corresponds to one selected data source. For a given data source, the scores of each candidate gene were computed using the associated sub-model. Then we ranked the candidate genes in each selected data source. Obviously, the most promising candidate genes would be at the front. At last, we integrated all the candidate genes rankings correspond to the given data sources and used an order statistics to obtain a single global ranking for mining the optimal disease risk genes. Based on the above strategies, we firstly collected 38 AML known genes as training gene set, in which 34 genes were from the Online Mendelian Inheritance in Man (OMIM) database, the other 4 AML known genes were verified by at least three published studies in genetic association database (GAD) database (Supplementary Table 1; All the 3 supplementary tables could be found in the website:http://www.bioapp.org/research/AMLhaplotype). Then up to 42 available data sources were used to train features and construct models, which generally contained the following categories: gene and protein function, chemical information, bio-molecular pathways, phenotypic information, interaction networks, expression profiles, expression ontologies, sequence features [8]. For instance, the ‘Gene and protein function’ category included resources such as Gene Ontology, Pfam, UniProt and InterPro. We could obtain the ranking of the candidate genes associated with each given data source. Then we fused all of these rankings from the separate data sources into a single ranking and obtained a global prioritization using order statistics [39]. The global order statistic Q-value was modeled by the Gamma-distribution, and then an approximate P-value was calculated according to the cumulative distribution function. The P-value represented the significance of this combination of rankings. If one candidate gene had a smaller P-value, it was regarded as more similar to the known AML genes and more likely to be considered as an AML risk gene [40]. In this study, the comprehensive prioritization results of candidate genes could be obtained from the online software Endeavour (https://endeavour.esat.kuleuven.be/Default.aspx) [8].

## SUPPLEMENTARY MATERIALS AND TABLES








