# Supplementary material for: Genome-wide haplotype association study identify the FGFR2 gene as a risk gene for Acute Myeloid Leukemia
Source: Oncotarget. 2016 Nov 26;8(5):7891–9. doi: 10.18632/oncotarget.13631 (PMC5352368; doi:10.18632/oncotarget.13631)
Supplement: Supplementary file 1 [file oncotarget-08-7891-s001.pdf]

Genome-wide haplotype association study identify the FGFR2 gene as a risk gene for Acute Myeloid Leukemia

SUPPLEMENTARY TABLES

Supplementary Table 1: 38 known AML susceptibility genes

See Supplementary File 1

Supplementary Table 2: 1754 significant haplotypes for AML

See Supplementary File 2

Supplementary Table 3: Detailed results of all candidate genes prioritization

See Supplementary File 3
